# Leveraging point-of-view camera and MediaPipe for objective hyperactivity assessment in preschool ADHD

**DOI:** 10.3389/fpsyt.2026.1769322

**Published:** 2026-03-04

**Authors:** Hakan Kayış, Çınar Gedizlioğlu

**Affiliations:** 1 Department of Child and Adolescent Psychiatry, Faculty of Medicine, Zonguldak Bülent Ecevit University, Zonguldak, Türkiye; 2 Department of Computer Engineering, İzmir University of Economics, İzmir, Türkiye

**Keywords:** ADHD, digital phenotyping, early screening, ecological validity, hyperactivity, machine learning, point-of-view video, pose estimation

## Abstract

**Background:**

Attention-Deficit/Hyperactivity Disorder (ADHD) often emerges in early childhood, with hyperactivity and impulsivity constituting the most prominent symptoms during the preschool period. Current assessment approaches rely largely on clinical interviews and behavior rating scales, which are susceptible to subjectivity and contextual bias. Objective, ecologically valid, and low-burden methods for quantifying hyperactivity in preschool settings remain limited.

**Methods:**

This observational, cross-sectional study investigated whether movement-based features extracted from teacher-worn point-of-view (POV) video recordings could differentiate preschool children at risk for ADHD-related hyperactivity from non-hyperactive peers. Fifty-one preschool children (48–60 months) participated in a standardized, three-minute storytelling interaction conducted in a familiar classroom environment. Video recordings were processed using MediaPipe pose estimation to derive region-specific movement indices across multiple body segments. Group differences were examined using statistical analyses. In addition, supervised machine learning models were applied to evaluate classification performance based on movement features.

**Results:**

Children in the hyperactivity-risk group exhibited significantly greater movement across several body regions, particularly distal upper- and lower-limb segments, compared to non-hyperactive peers. Machine learning analyses indicated promising classification performance, with the support vector machine achieving an accuracy of 84.31%, sensitivity of 80.0%, specificity of 87.10%, and an area under the receiver operating characteristic curve (AUC) of 0.83. Permutation-based feature importance analyses highlighted distal limb movements as the most informative features for classification.

**Conclusions:**

These findings suggest that POV-based, vision-driven assessment provides a promising, objective, and ecologically valid approach for quantifying hyperactivity-related motor behavior in preschool children. While not intended as a standalone diagnostic tool, this low-burden framework may serve as a valuable complement to existing screening practices and support early identification efforts in educational settings.

## Introduction

Attention-Deficit/Hyperactivity Disorder (ADHD) is a neurodevelopmental condition characterized by inattention, hyperactivity, and impulsivity, with onset typically in early childhood (1). It is associated with impairments in academic, social, and daily functioning (2–4). Although prevalence estimates vary, global childhood prevalence is generally around 7% (5), with recent U.S. data reporting 10.5% among children aged 3–17 years (6). Preschool prevalence rates are estimated between 3% and 10.5%, depending on diagnostic methods and cultural contexts (7–9). Symptom presentation also shifts with development, with hyperactivity/impulsivity predominating in younger children and inattention becoming more prominent with age (10).

The diagnosis of ADHD is currently based on a comprehensive clinical evaluation involving the child, parents, and teachers, with reference to DSM-5 criteria (11). Criteria require that symptoms persist for at least six months, are present in more than one context, and cause functional impairment (1). In preschoolers, inattentive symptoms may be less distinctive developmentally, while hyperactivity, impulsivity, difficulties with rule-following, and problems in social adaptation are particularly salient (10, 12). To support diagnostic decision-making, standardized scales such as the Conners Early Childhood Parent and Teacher Rating Scales, the ADHD Rating Scale-IV Preschool Version (ADHD-RS-IV-P), and the Early Childhood Inventory-4 (ECI-4) are widely used (13–15). These tools provide valuable reports from parents and teachers regarding children’s behavior across settings.

Despite their clinical utility, there is currently no single biological marker or objective diagnostic test for ADHD. Symptom rating scales and clinical judgment remain central to ADHD assessment; however, current diagnostic pathways rely heavily on clinical interviews and multi-informant rating scales that are vulnerable to recall and expectancy biases (16). In response, a range of objective measures has been investigated. Chief among these are Continuous Performance Tests (CPTs). Computerized paradigms that index sustained attention, response inhibition, and vigilance through target–nontarget discrimination and speed–accuracy trade-offs; typical outcome metrics include omission and commission errors, reaction time, and reaction time variability (17). Notably, neurocognitive tests have also been administered in some studies with preschool-aged children (18, 19).

Neurophysiological approaches have likewise been explored. Electroencephalography (EEG) has been recorded both at rest and during CPT performance, and several studies have applied machine-learning techniques to EEG features to aid ADHD classification (20, 21). Parallel efforts using neuroimaging—such as applying machine learning to features derived from functional MRI—have also been reported (22). More recently, virtual reality (VR) paradigms that simulate everyday tasks and capture ecologically valid behavioral and performance data have emerged as a promising direction (23). Nevertheless, these technologies, whether behavioral, neurophysiological, or neuroimaging-based, are not yet sufficiently reliable to serve as standalone diagnostic methods (24).

In addition to these task-based cognitive measures and brain-based methods, real-world behavioral activity has been quantified using both wearable sensors and computer vision methods. Accelerometers provide flexibility across settings but require long monitoring periods and extensive data processing to ensure reliability (25, 26). Marker-based systems such as Infrared Motion Tracking (IMT) capture movements through reflective markers and infrared cameras (27). More recently, video-based methods have shown considerable promise. For example, Wehrmann and Müller (2015) estimated activity levels using webcam video compression, while Chiu et al. (2024) applied pixel subtraction and machine learning techniques to clinical recordings to distinguish children with ADHD from controls (28, 29). Yet, even when these methods operate in “naturalistic” environments, they often impose nontrivial burdens—children may need to wear devices (23, 25) or interact with conspicuous hardware (28).

To address these limitations, point-of-view (POV) eyeglasses offer an unobtrusive, ecologically valid means of capturing moment-to-moment behaviors without materially disrupting natural interaction. In psychiatry, POV systems have been applied across a range of conditions and modalities, particularly within treatment interventions. For example, they have been used as supportive tools to facilitate socialization in autism (30, 31); and as instructional media for teaching play skills to autistic children (32). Beyond intervention, POV cameras have been shown to measure eye contact during natural social exchanges in a safe and valid manner, while offering a relatively low-cost, scalable approach (33). More recently, Ahn et al. (2024) leveraged POV-derived video to quantify gaze and smiling behaviors and examined their associations with autism severity, further underscoring the promise of POV methodologies for objective assessment (34). Extending the use of POV approaches beyond developmental populations, Kayış et al. (2025) employed multimodal features derived from POV recordings during semi-structured clinical interviews to detect autism and depression in two related studies, highlighting the ecological validity of first-person behavioral sensing in psychiatric research (35, 36). However, existing POV work has focused primarily on affective behaviors, social attention and intervention; to our knowledge, no studies have quantitatively evaluated preschool hyperactivity in naturalistic interactions using POV systems.

Motivated by the unmet need for cost-effective, developmentally appropriate, and objective behavioral measures for preschool populations, we leverage first-person, point-of-view (POV) recordings. We capture a standardized, teacher-led storytelling interaction and derive quantitative behavioral indices directly from the classroom—an ecologically valid, real-life context. To our knowledge, this is the first investigation to objectively characterize preschool hyperactivity using POV eyeglasses and to anchor evaluation in authentic social exchanges while maintaining a scalable, resource-efficient workflow.

We address two questions:

1. In naturalistic classroom interactions, do movement-based features derived from body regions (e.g., head, trunk, upper limbs) differ between preschoolers at risk for ADHD and non-risk peers—i.e., do whole-body mobility metrics extracted from in-class POV video show meaningful group differences?
2. Can these differences be detected and reliably classified using machine-learning methods suitable for screening, yielding models that are performant, interpretable where possible?

## Methods

### Study design

This study employed an observational, cross-sectional design to quantify hyperactivity-related behavioral markers in preschool children during a brief, standardized story-listening interaction. A single trained preschool teacher (female, ~32 years) conducted all sessions while wearing point-of-view (POV) glasses with a front-facing camera (1920×1080, 30 fps), enabling unobtrusive, first-person recording of child behavior from a natural classroom perspective. Each child was seen individually in a quiet, vacated classroom within their own school; lighting and seating geometry were held constant, with the teacher and child positioned face-to-face at a fixed distance of 300 cm. The teacher delivered a three-minute story via headphones while reading aloud to preserve natural prosody. Immediately afterward, a six-item, segment-aligned comprehension questionnaire was administered to index attention and recall. POV videos were de-identified and processed offline by an engineer blinded to diagnostic grouping, using a predefined computer-vision pipeline (MediaPipe pose estimation) to extract frame-wise landmarks and derive movement metrics (e.g., cumulative displacement, landmark-based movement variability) as objective proxies of hyperactivity.

### Recruitment

Our study was conducted in a regional preschool. Ethical approval was obtained from the Non-Interventional Research Ethics Committee of Zonguldak Bülent Ecevit University (decision no. 2025/07, dated 09/04/2025). Permission to conduct the study at the participating preschool was obtained through the Turkish Ministry of National Education. Written informed consent was obtained from the parents or legal guardians of all participating children. A total of 51 preschoolers participated in the study (29 boys, 22 girls), aged between 48 and 60 months. Participant recruitment and data collection were conducted between May and September 2025. All children were evaluated by an experienced child and adolescent psychiatrist using DSM-5-TR criteria, with additional information obtained through structured teacher interviews to assess classroom behavior.

Exclusion criteria were current medication use, intellectual disability, autism spectrum disorder, and severe psychiatric comorbidities other than ADHD. Accordingly, 2 children were excluded due to cognitive impairment, 3 children due to anxiety disorder, 1 children due to autism spectrum disorder. Teachers completed the Conners Teacher Rating Scale–Revised: Short Form (CTRS-R:S), a widely used instrument for assessing hyperactivity, inattention, and oppositional behaviors in early childhood settings. The scale has demonstrated robust psychometric properties in international preschool samples, including strong internal consistency and factor validity (37, 38). The Turkish adaptation has likewise shown high internal consistency and a factor structure consistent with the original version, supporting its applicability for evaluating teacher-reported behavioral difficulties in Turkish children (39). However, although the Turkish version has established linguistic and psychometric adequacy, nationally normed T-scores and culture-specific clinical cut-offs have not yet been developed. In the present study, CTRS-R:S Hyperactivity scores were therefore used as dimensional indices to characterize symptom severity and to examine their associations with movement-based metrics derived from POV video.

The hyperactivity-risk group consisted of preschoolers classified as positive for ADHD-related hyperactivity/impulsivity according to DSM-5-TR criteria, indicating developmentally inappropriate levels of hyperactive and/or impulsive symptoms. Classroom manifestations of hyperactivity were further corroborated through structured teacher reports, including persistent difficulty remaining seated, excessive motor activity, and disruptive movement during group activities. The non-hyperactive comparison group included children for whom evaluation according to DSM-5-TR criteria did not indicate clinically significant hyperactivity, impulsivity, or functional impairment, and whose teachers reported no concerns regarding activity level or behavioral regulation in the classroom. Using these criteria, the final sample comprised 51 preschool children aged 48–60 months, including 20 children in the hyperactivity-risk group (14 boys and 6 girls) and 31 children in the non-hyperactive comparison group (16 girls and 15 boys). Children for whom the DSM-5-TR–based evaluation and teacher-reported classroom behavior were inconsistent (n = 7) were excluded *a priori* from group assignment to ensure classification based on concordant behavioral evidence.

### Clinical experiment

A single preschool teacher (female, 32 years) conducted all recordings. This design choice was intended to minimize systematic variability related to interaction style and head-mounted POV camera motion, and to reduce the likelihood that between-group differences could be attributed to experimenter-related factors rather than child behavior. Before data collection, she underwent structured training in the use of point-of-view (POV) glasses and completed pilot sessions to ensure protocol fidelity, with emphasis on maintaining a stable head position and centering the child in the field of view. To minimize schedule-related variability, sessions were held during standardized morning activity periods. All assessments took place in one preschool, in the same vacated classroom, under constant environmental conditions (fixed teacher–child distance and consistent ambient lighting, with no background noise).

**Figure 1:**
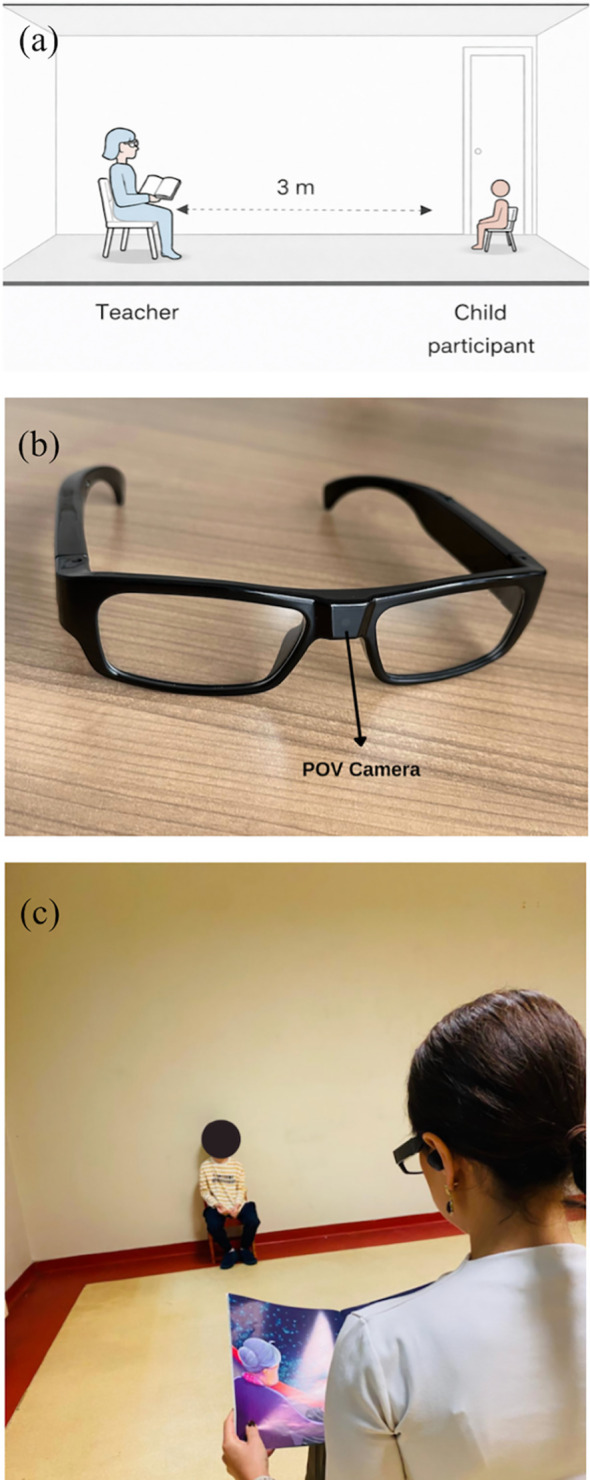
Experimental setup and configuration of the recording environment. **(a)** Schematic diagram illustrating the spatial geometry of the testing room, with the teacher and child participant seated face-to-face at a distance of 3 m **(b)** Photograph of the first-person point-of-view (POV) wearable camera used for video recording. **(c)** Photograph of the actual experimental setting, demonstrating the nature of the recording setup during the teacher–child interaction.

**Figure 2:**
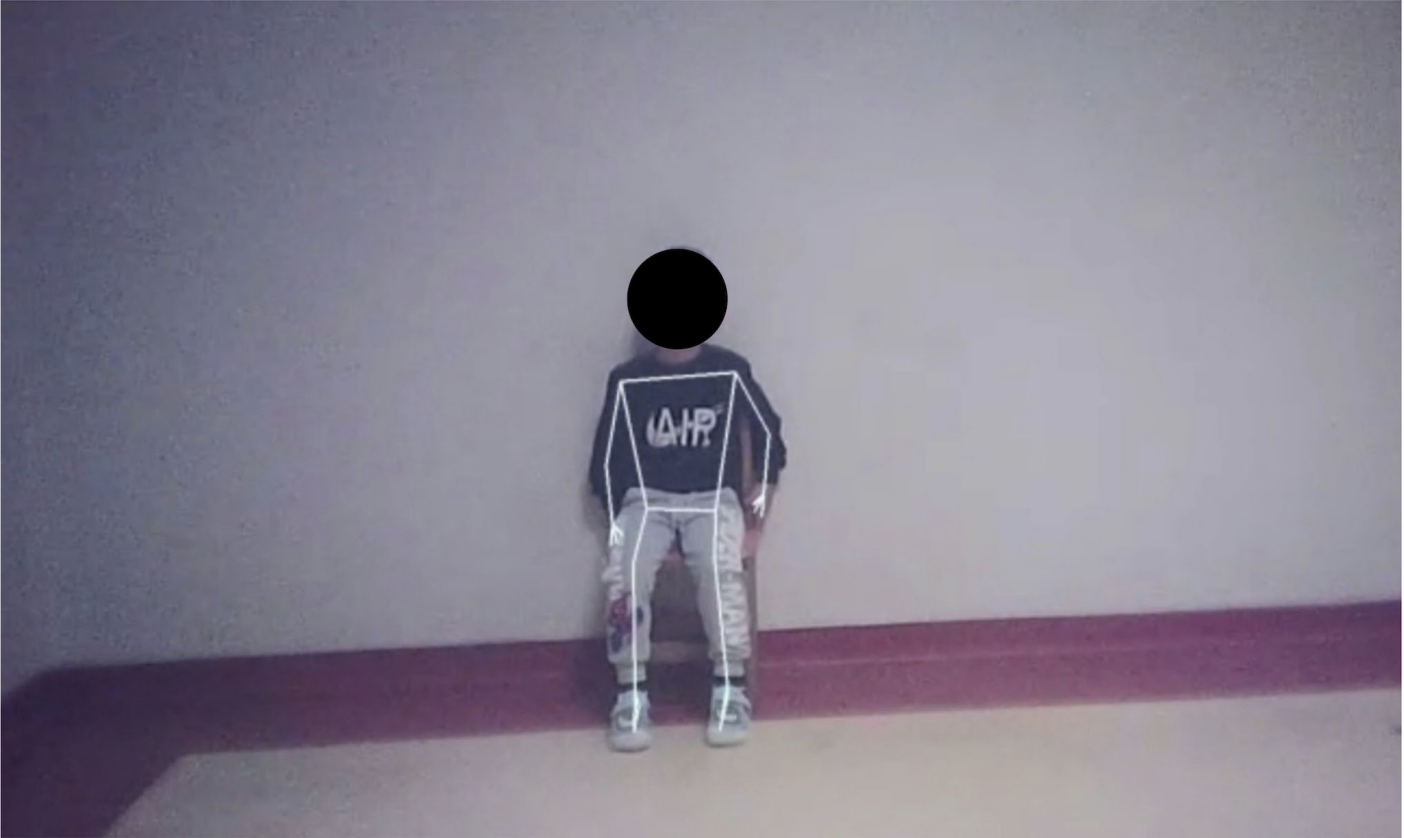
Representative POV camera frame showing the child seated opposite the teacher and the corresponding MediaPipe Pose landmark detection used for automated movement analysis. For privacy reasons, the participant’s face is fully masked and facial landmarks are not shown. The figure is provided for illustrative purposes only.

Each child was individually brought into the room and informed that a story would be read aloud. The teacher and child sat face-to-face at a fixed distance of 300 cm. The teacher wore POV glasses and tell the story for exactly three minutes while simultaneously listening through headphones to standardize delivery. A storybook was placed in front of the teacher to maintain the naturalness of the interaction (**Figure 1**). Video was captured at 1920 × 1080 resolution and 30 frames per second (33). The POV recordings provided a continuous first-person view of the child during the interaction. These videos were subsequently processed using the MediaPipe Pose framework to extract body landmarks and quantify movement patterns. **Figure 2** illustrates a representative POV video frame and the corresponding pose landmark detection used for movement analysis.

For the procedure, the teacher read the story entitled *“The Journey Game”* (*Yolculuk Oyunu*) to each participant for a duration of three minutes. This story was distributed to preschools in the 2024–2025 academic year by the Turkish Ministry of National Education (40). Immediately after the session, the researcher administered a structured questionnaire related to the story, consisting of six comprehension questions corresponding to each 30-second segment (**Appendix 1**).

The comprehension questions were selected to correspond directly to each 30-second segment of the story, ensuring an age-appropriate cognitive load. The story’s official distribution by the Ministry of National Education guaranteed developmental suitability and standardization across participants. This design supports content validity, as all children were exposed to the same stimuli and evaluated with identical questions, targeting attention, recall, and comprehension processes that are particularly relevant for hyperactivity and inattention.

### Feature extraction

Data collected during the standardized interviews were subsequently analyzed using computer software. Recordings were temporally aligned to the onset of the teacher’s first spoken word and trimmed to an exact 180-s window (≈5,400 frames at 30 fps). Clips were reviewed for drop-frames and timing jitter. Tracking quality was defined per frame and per landmark using the model’s confidence output; frames with landmark confidence <0.50 were marked invalid for that landmark. Sessions were excluded if global missingness exceeded 20% of frames (n=3). For sessions with 0–20% missing data, we applied a two-tiered procedure: (i) short gaps ≤10 consecutive frames (≤333 ms) were filled by linear interpolation in 3D for the affected landmark; (ii) longer gaps were smoothed with a constant-velocity Kalman filter (41) initialized on the nearest valid samples. In addition, landmark trajectories were temporally smoothed using the One Euro Filter, an adaptive low-pass filter that reduces high-frequency jitter while preserving rapid, meaningful movements (42).

**Figure 3:**
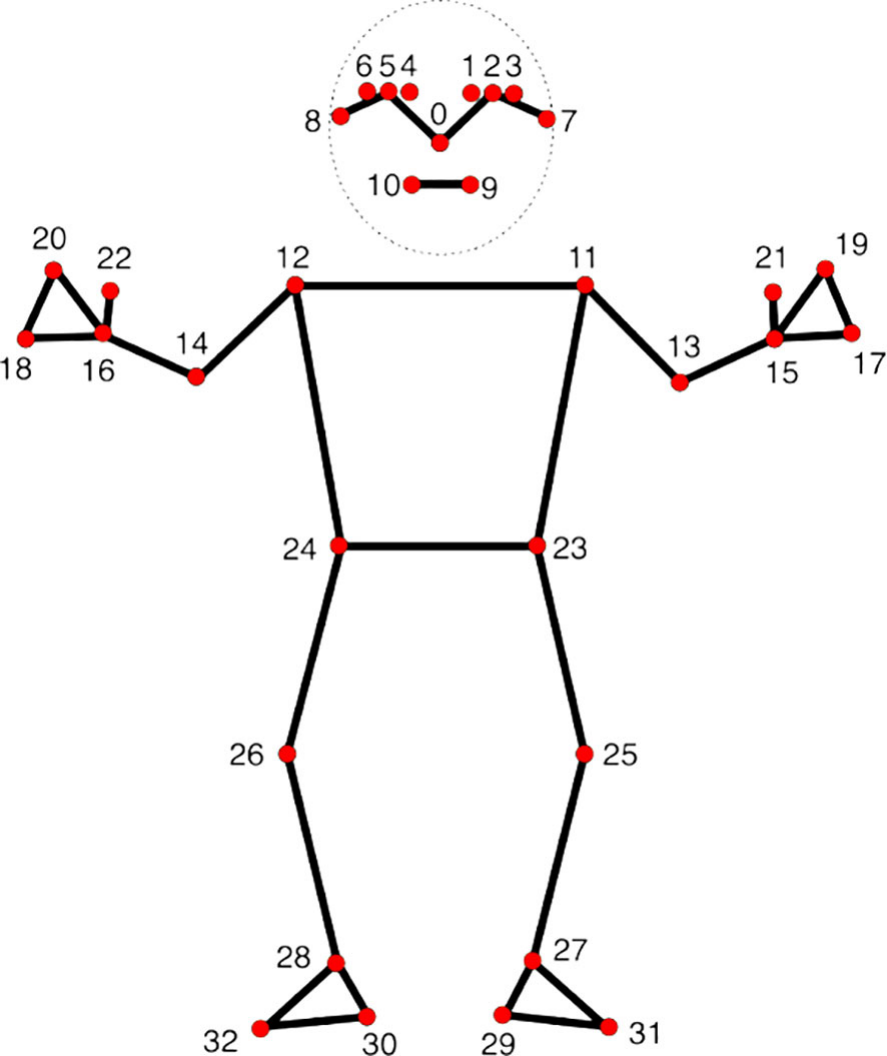
MediaPipe Pose Landmarks used in the present study (33 keypoints) (43). The figure illustrates the 33 anatomical landmarks detected by the MediaPipe Pose Landmarker model, including facial keypoints (0–10), upper-body landmarks (11–24), and lower-body landmarks (25–32). These landmarks were extracted frame-by-frame from POV recordings and used to compute movement-based features (e.g., cumulative displacement, segment-wise variability) as objective indicators of child motor activity during the storytelling task.

Video recordings were processed with MediaPipe, an open-source real-time computer vision framework developed by Google for human pose estimation (43). Prior research has shown that MediaPipe provides reliable algorithms for motion tracking and joint angle estimation with acceptable error margins compared to gold standard marker-based systems, making it a cost-effective tool for clinical and developmental applications (44–46). Importantly, the framework has also been applied successfully in studies involving infants (47) and children across diverse age groups (48). For each frame, the MediaPipe Pose Landmarker returned 3D coordinates (x, y, z) for 33 anatomical keypoints (**Figure 3**).

Although the MediaPipe Pose Landmarker outputs 3D coordinates (x, y, z), the depth (z-axis) estimates derived from monocular RGB video are known to be relative and less reliable in absolute terms, particularly in single-camera conditions (49). Accordingly, movement quantification in the present study focused primarily on displacements within the image plane (x–y), which provide the most robust and interpretable estimates of gross motor activity. Z-axis displacements were therefore not included in the primary movement indices and were considered only as approximate indicators of forward–backward motion relative to the camera.

After extraction, landmarks were aggregated into anatomically meaningful composite points to reduce redundancy and improve tracking robustness. Specifically, the head was represented by the centroid of 11 facial landmarks (nose, eyes, ears, and mouth), which move largely as a rigid unit during gross head displacement. Similarly, hand proxies were computed as the centroid of three distal hand landmarks (thumb, index, and pinky) for each side, and foot proxies were computed as the centroid of the heel and foot-index landmarks for each side. Subsequent analyses were performed on the resulting set of composite and joint landmarks: head (11-landmark centroid), left/right shoulder, left/right elbow, left/right wrist, left/right hip, left/right knee, left/right ankle, left/right hand (3-landmark centroid), and left/right foot (2-landmark centroid). Movement was quantified by computing Euclidean displacement over short temporal windows. Specifically, landmark positions were averaged within consecutive non-overlapping 5-frame windows (≈167 ms at 30 fps), and displacement was calculated between successive window means. These displacements were then summed across the 3-minute recording to derive cumulative movement indices reflecting overall motor activity during the storytelling task. This analytic strategy reduces the influence of frame-level jitter inherent in monocular pose estimation and captures behaviorally meaningful motor fluctuations. The approach was conceptually inspired by displacement-based methods used in prior work to quantify naturalistic movement dynamics from automatic pose estimation (50).

In accordance with prior work, movement quantification was performed using a pelvis-root reference coordinate frame, with the root joint positioned at the center of the pelvis (51, 52). Person-centric (root-centric) representations express all 3D keypoint coordinates relative to this central root, effectively re-anchoring the body so that joint positions are computed with respect to the pelvis. This approach is particularly advantageous in single-person, monocular pose estimation, as it reduces scale variation and stabilizes body-motion trajectories (53). By using a pelvis-centered reference frame, movement features primarily captured relative limb and segmental motion rather than absolute whole-body translation in the scene.

For each participant, the following parameters were extracted:

- Regional activity indices were computed as cumulative displacement measures for each anatomically defined body region, including the head (11-landmark centroid), left and right shoulders, elbows, wrists, hips, knees, ankles, distal hands, and distal feet.
- The global activity index was calculated as the sum of all regional activity indices, providing an integrated measure of overall motor activity across the entire body during the 3-minute storytelling session.

These quantitative features were then aggregated for subsequent statistical analysis and machine learning models aimed at differentiating children with elevated hyperactivity risk from their non-hyperactive peers.

### Statistical analysis

Statistical analyses were conducted using IBM SPSS Statistics (Version 27). Group differences in demographic variables were examined using independent-samples *t* tests for age and chi-square tests for sex. For movement-based features, normality was assessed using the Shapiro–Wilk test. Depending on distributional characteristics, between-group comparisons were performed using independent-samples *t* tests or Mann–Whitney *U* tests. We calculated effect sizes for all univariate group comparisons. For Mann–Whitney U tests, effect size was expressed as r and derived from the standardized test statistic and the total sample size. Associations between regional activity indices, the global activity index, and teacher-rated hyperactivity scores were examined using Spearman correlation coefficients. Statistical significance was set at p < 0.05 (two-tailed).

Because multiple, conceptually related motor activity outcomes were examined, we adopted a two-tiered approach to control for multiple comparisons.

As the primary correction strategy, false discovery rate (FDR) control was applied across all univariate movement features included in the main univariate analyses using the Benjamini–Hochberg procedure (q = 0.05).

In addition, for anatomically structured sensitivity analyses, movement features were grouped *a priori* into five theoretically and anatomically coherent domains, and family-wise error rate control was performed at the domain level using Bonferroni correction.

- Head domain, represented by a composite head movement index derived from facial landmarks (mhead = 1);
- Right upper limb domain, including right shoulder, right elbow, right wrist, and right hand movement indices (mRUL= 4);
- Left upper limb domain, including left shoulder, left elbow, left wrist, and left hand movement indices (mLUL = 4);
- Right lower limb domain, including right hip, right knee, right ankle, and right foot movement indices (mRLL = 4);
- Left lower limb domain, including left hip, left knee, left ankle, and left foot movement indices (mLLL = 4).

Within each domain, Bonferroni correction was applied to adjust for multiple comparisons, with the domain-specific significance threshold defined as α = 0.05 divided by the number of variables within that domain. Accordingly, the adjusted significance level was set at α = 0.0125 for each upper- and lower-limb domain. No correction was applied to the head domain, which comprised a single outcome measure. The global activity index was treated as the primary outcome and was therefore evaluated without correction.

### Machine learning analysis

In addition to group-level statistical comparisons, machine learning approaches were employed to evaluate the discriminative performance of movement-based behavioral features in distinguishing preschool children at risk for ADHD-related hyperactivity from non-hyperactive peers. The feature set consisted of region-specific movement indices derived from pose-based analysis, including the head, bilateral shoulders, elbows, wrists, distal hands, knees, ankles, and distal feet. Hip landmarks were not included as independent features, as movement was quantified relative to a pelvis-based reference frame. The global activity index was also excluded from machine learning analyses to avoid redundancy and potential information leakage, given that it represents a composite of regional movement measures.

Classification performance was quantified using a set of complementary metrics, including accuracy, precision, recall (sensitivity), specificity, F1-score, and the area under the receiver operating characteristic curve (ROC–AUC), enabling a robust and multifaceted evaluation of model performance.

Given the tabular structure of the extracted features and the modest sample size, several supervised learning algorithms with complementary strengths were selected. Tree-based ensemble methods—including Random Forests (54), Extremely Randomized Trees (55), and AdaBoost with decision trees as base estimators (56)—were chosen due to their robustness to feature scaling, ability to model nonlinear relationships, and suitability for small to medium-sized datasets. In addition, Support Vector Machines (SVM) and k-Nearest Neighbors (KNN) classifiers were evaluated, as these distance- and geometry-based methods are well suited for continuous movement features defined within a common coordinate space. For each model, hyperparameters were optimized independently to maximize classification performance.

To mitigate overfitting and obtain an unbiased estimate of model generalization performance, a nested cross-validation scheme was employed for all machine learning models. In this framework, an outer cross-validation loop was used to evaluate model performance, while an inner cross-validation loop within each training fold was applied for hyperparameter optimization. Hyperparameters were selected based on validation performance in the inner loop and subsequently evaluated on held-out test data in the outer loop. Final performance metrics were obtained by averaging results across the outer cross-validation folds. This nested procedure reduces the risk of optimistic bias that can arise when hyperparameters are tuned and evaluated on the same data and is particularly recommended for studies with limited sample sizes.

In the nested cross-validation framework, both the inner (hyperparameter optimization) and outer (performance evaluation) loops employed a stratified 5-fold cross-validation scheme. In each iteration, models were trained on four folds and evaluated on the remaining fold, ensuring that all observations contributed to both training and validation across folds. Performance metrics were averaged across the outer folds to obtain a robust estimate of generalization performance. The use of 5-fold cross-validation provides a favorable trade-off between bias and variance, allowing efficient use of available data while maintaining computational efficiency and stability compared to leave-one-out cross-validation or a single train–test split.

Another rationale for selecting tree-based ensemble methods was their inherent robustness to overfitting. By aggregating predictions from multiple weakly correlated decision trees, these models reduce variance and improve generalization performance, particularly in small to moderate-sized datasets.

In the implementation of the Support Vector Machine (SVM) and k-Nearest Neighbors (KNN) classifiers, class imbalance was explicitly addressed through a weighting strategy. Specifically, class weights were assigned inversely proportional to the number of samples in each class, such that minority classes received higher weights and majority classes received lower weights. This approach ensured that misclassification of underrepresented classes incurred a greater penalty during model training, thereby mitigating bias toward majority classes and improving the models’ ability to learn from imbalanced data distributions.

## Results

### Participant characteristics

The final sample consisted of 51 preschool children aged 48–60 months, including 20 children in the hyperactivity-risk group and 31 children in the non-hyperactive comparison group. The groups did not differ in age or sex distribution (**Table 1**).

**Table 1:** Demographic characteristics of the study sample.

Variable	Control (n = 31)	Hyperactivity-risk (n = 20)	Test	P
Age (months), M (SD)	52.58 (3.40)	52.75 (3.95)	t(49) = −0.16	0.87
Sex (boys/girls)	15/16	14/6	χ²(1) = 2.32	0.13

### Task-related performance

The two groups did not differ significantly in their performance on the story-related comprehension questions. The control group achieved a mean of 2.55 correct responses (SD = 1.36), while the hyperactivity-risk group achieved a mean of 2.20 (SD = 1.32). This difference was not statistically significant (p = 0.37).

### Conners hyperactivity scores and their associations with movement indices

Children in the non-hyperactive comparison group showed low Conners hyperactivity scores (mean = 1.52, SD = 2.86; median = 0), whereas higher scores were observed in the hyperactivity-risk group (mean = 10.80, SD = 6.58; median = 11). Given non-normality in the non-hyperactive comparison group, we used nonparametric analysis. A Mann–Whitney U test indicated a significant group difference, with higher hyperactivity scores observed in the hyperactivity-risk group (U = 65.50, p < 0.001).

We conducted Spearman correlation analyses to examine associations between regional movement indices and teacher-rated hyperactivity scores. Left ankle movement showed a positive correlation with hyperactivity scores (ρ = 0.32, 95% CI [0.05, 0.56], p = 0.022). Similar patterns were observed for right ankle movement (ρ = 0.28, 95% CI [0.03, 0.51], p = 0.046) and left distal hand movement (ρ = 0.28, 95% CI [0.04, 0.51], p = 0.049). No other regional movement indices showed statistically significant correlations with hyperactivity ratings (**Table 2**). Given the exploratory nature of these analyses and the modest sample size, correlation results were not corrected for multiple comparisons. Accordingly, findings are interpreted cautiously, with greater emphasis on the direction of effects rather than on strict statistical significance.

**Table 2:** Spearman correlations between regional movement indices and teacher-rated hyperactivity scores.

Movement region	Spearman’s ρ	P value
Head	0.22	0.117
Left shoulder	0.11	0.431
Right shoulder	0.13	0.359
Left elbow	0.20	0.154
Right elbow	0.18	0.200
Left wrist	0.26	0.066
Right wrist	0.18	0.198
Left knee	0.26	0.063
Right knee	0.18	0.218
**Left ankle**	**0.32**	0.022*
**Right ankle**	**0.28**	0.046*
**Left distal hand**	**0.28**	0.049*
Right distal hand	0.19	0.175
Left distal foot	0.26	0.066
Right distal foot	0.23	0.111

Bold values indicate statistically significant results.

### Group differences in movement indices

We assessed the normality of regional and global movement indices separately for each group. Across the 17 regional indices and the global activity index, most variables showed significant deviations from normality in at least one group (p < 0.05). Given violations of normality assumptions, we applied nonparametric statistical methods.

We observed significant group differences across multiple movement indices, with consistently higher activity levels in the hyperactivity-risk group. Differences were most pronounced in distal upper- and lower-limb regions, whereas proximal regions showed weaker or non-significant effects. Head movement also differed significantly between groups. This overall pattern was preserved after false discovery rate (FDR) correction (**Table 3**).

**Table 3:** Group differences in regional movement indices between the hyperactivity-risk group and the non-hyperactive comparison group.

Body region/Feature	Test statistic (U)	r value	Raw p-value	FDR-adjusted p-value	Direction
Head	182.00	0.346	0.014	0.021	Hyperactivity-risk > Control
Right shoulder	224.00	0.233	0.097	0.105	n.s.
Left shoulder	226.00	0.227	0.105	0.104	n.s.
Right elbow	165.00	0.392	0.005	0.011	Hyperactivity-risk > Control
Left elbow	162.00	0.400	0.004	0.012	Hyperactivity-risk > Control
Right wrist	183.00	0.345	0.014	0.020	Hyperactivity-risk > Control
Left wrist	163.00	0.398	0.005	0.010	Hyperactivity-risk > Control
Right distal hand	186.00	0.335	0.017	0.021	Hyperactivity-risk > Control
Left distal hand	160.00	0.405	0.004	0.010	Hyperactivity-risk > Control
Right knee	197.00	0.305	0.029	0.033	Hyperactivity-risk > Control
Left knee	184.00	0.340	0.015	0.021	Hyperactivity-risk > Control
Right ankle	118.00	0.518	<0.001	0.001	Hyperactivity-risk > Control
Left ankle	124.00	0.503	<0.001	0.001	Hyperactivity-risk > Control
Right distal foot	143.00	0.451	0.001	0.005	Hyperactivity-risk > Control
Left distal foot	146.00	0.442	0.002	0.007	Hyperactivity-risk > Control

Differences were assessed using Mann–Whitney U tests. Effect sizes are reported as r. False discovery rate (FDR)–adjusted p-values are shown. Positive effects indicate higher movement levels in the hyperactivity-risk group.

Domain-based Bonferroni-corrected analyses revealed significant group differences in multiple distal movement indices, with higher activity levels in the hyperactivity-risk group. In the upper limbs, significant differences were observed for right elbow (p = 0.005), left elbow (p = 0.004), left wrist (p = 0.005), and left distal hand movements (p = 0.004). Shoulder movements, right wrist and right distal hand did not reach statistical significance. In the lower limbs, significant group differences were found for right ankle (p < 0.001), left ankle (p < 0.001), right distal foot (p = 0.001) and left distal foot (p = 0.002). Knee movements did not consistently remain significant after correction.

We observed a significant group difference in the global activity index, reflecting overall motor activity across all body regions during the 3-minute storytelling task. Preschoolers in the hyperactivity-risk group exhibited higher global motor activity than the non-hyperactive comparison group (Mann–Whitney U = 155.00, p = 0.003), with higher mean ranks in the hyperactivity-risk group (33.75) compared to controls (21.00).

### Inter-regional correlations of movement indices

**Figure 4:**
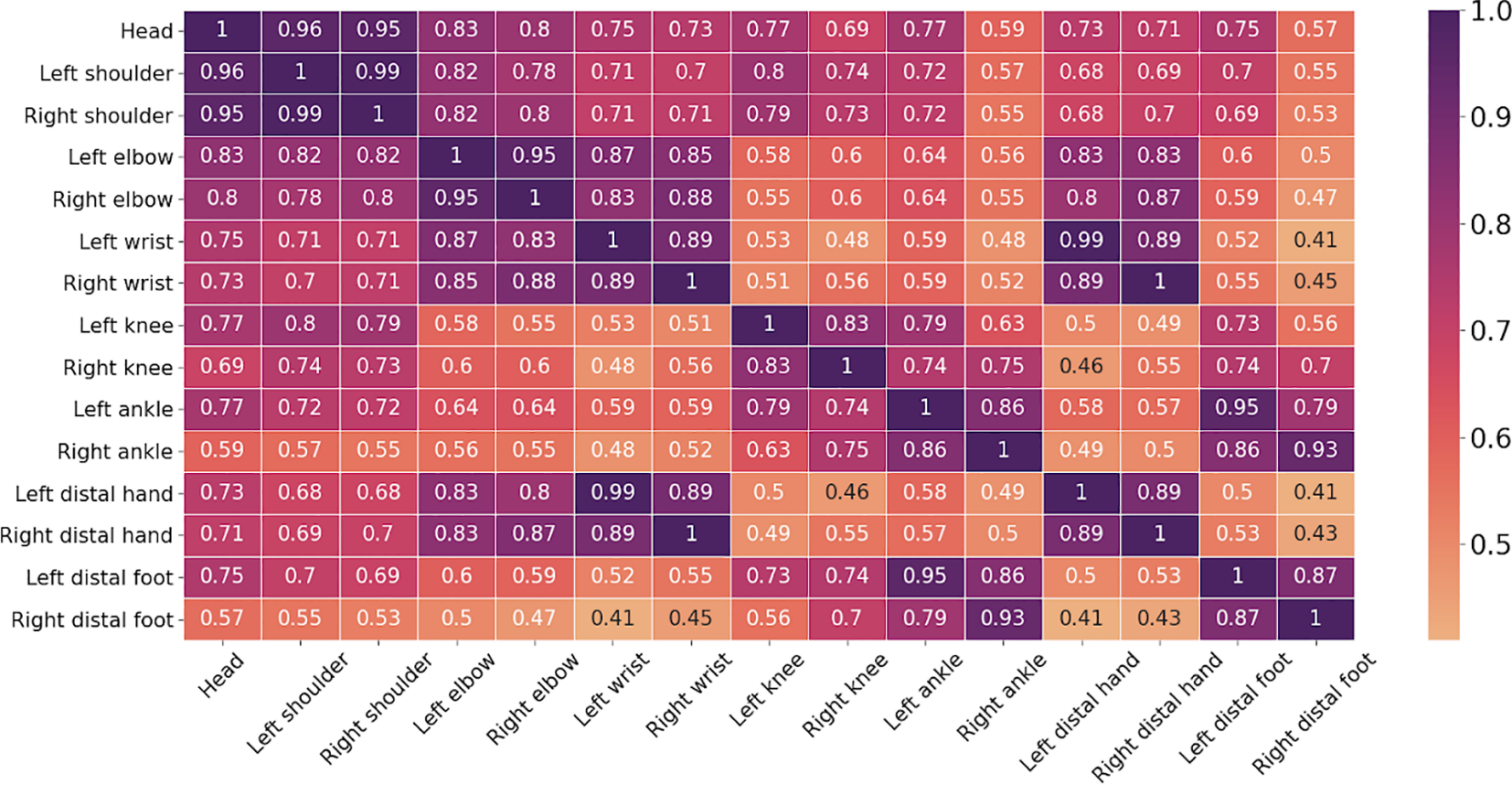
Inter-regional movement correlation heatmap. Spearman correlation coefficients illustrating pairwise associations between regional movement indices across all participants.

To further examine whether apparent right–left asymmetries in feature importance could be explained by shared movement variance across body regions, we computed inter-regional correlations among all movement indices. As shown in **Figure 4**, strong bilateral correlations were observed across homologous body segments.

### Machine learning classification results

Following statistical analyses, machine learning models were applied to further evaluate the discriminative ability of movement-based behavioral features in distinguishing preschool children at risk for ADHD-related hyperactivity from non-hyperactive peers. Multiple supervised classifiers were tested, including tree-based ensemble methods, k-nearest neighbors, and support vector machines (SVM). Among the evaluated models, the SVM demonstrated the highest and most consistent performance across evaluation metrics and was therefore selected as the primary model for reporting classification results.

**Figure 5:**
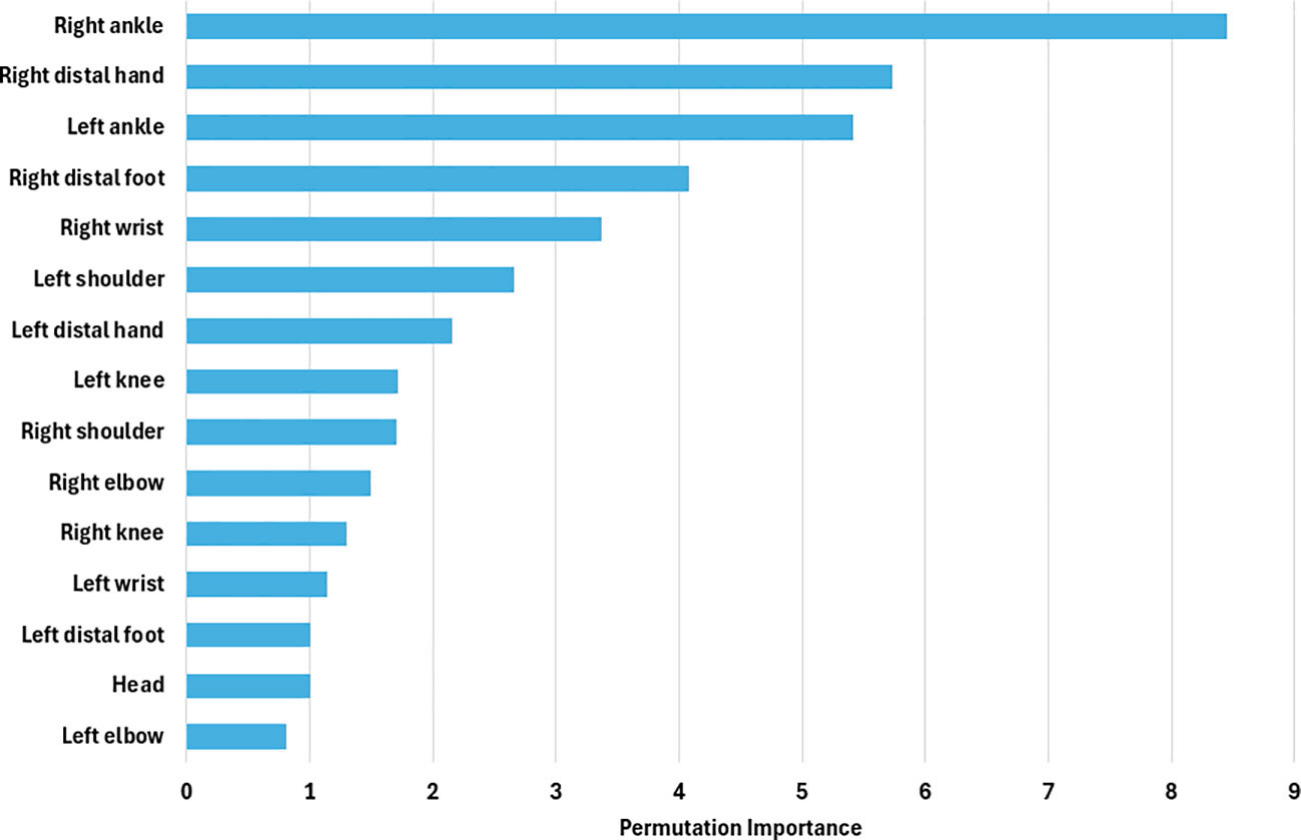
Permutation feature importance scores for movement-based features used in the support vector machine classifier. Higher values indicate features that contributed more strongly to distinguishing children in the hyperactivity-risk group from the non-hyperactive comparison group.

Feature importance estimates were computed and are reported in **Figure 5**. These computations were performed within the outer loop of the nested cross-validation procedure. Following completion of the inner cross-validation loop, feature importance was assessed for the best-performing estimator, defined as the model with the optimal set of hyperparameters.

Feature relevance was evaluated using permutation feature importance (PFI) (54). In this approach, the values of a single feature are randomly permuted across samples while all other features are kept unchanged, thereby disrupting the relationship between that feature and the outcome while preserving its marginal distribution. The trained model is then re-evaluated on the validation set. A substantial decrease in model performance following permutation indicates that the feature contributes meaningfully to prediction accuracy, whereas minimal change suggests limited predictive relevance.

For each feature, an importance score was calculated as the difference between the baseline model performance and the performance obtained after permutation. To enhance robustness, the permutation procedure was repeated multiple times and importance scores were averaged across repetitions. Larger positive values indicate greater feature importance, values close to zero reflect negligible contribution, and negative values suggest that a feature may introduce noise or adversely affect model performance. Taken together, movement features showing significant group differences and associations with teacher-rated hyperactivity severity (**Table 2**) largely overlapped with those contributing most strongly to the machine learning classifier, as reflected by permutation-based feature importance scores (**Figure 5**).

To further examine the discriminative capacity of the extracted movement-based features, machine learning models were evaluated using a separate 5-fold cross-validation procedure. Performance metrics reflected classification accuracy and are reported with Wilson 95% confidence intervals. Among the tested classifiers, the support vector machine (SVM) achieved the highest performance. The performance metrics of tested classifiers are presented in **Table 4**. Each performance metric is accompanied by Wilson 95% confidence intervals.

**Table 4:** Performance of machine learning models for distinguishing the hyperactivity-risk group from the non-hyperactive comparison group.

Model	Accuracy	Precision	Sensitivity	Specificity	F1 score	Mean CV-fold AUC (± SD)	Pooled out-of-fold AUC
Random Forest	70.59% (57.0–81.3)	60.87% (47.2–73.0)	70% (56.4–80.8)	70.97% (57.4–81.6)	65.12% (51.4–76.7)	0.86 ± 0.07	0.76 (0.62–0.90)
Extra Trees	64.71% (51.0–76.4)	54.55% (41.0–67.4)	60% (46.3–72.3)	67.74% (54.1–78.9)	57.14% (43.5–69.7)	0.84 ± 0.03	0.76 (0.62–0.90)
AdaBoost	66.67% (53.0–78.0)	57.14% (43.5–69.7)	60% (46.3–72.3)	70.97% (57.4–81.6)	58.54% (44.9–71.0)	0.77 ± 0.12	0.71 (0.56–0.86)
SVM	**84.31% (72.0–91.8)**	**80% (67.1–88.7)**	**80% (67.1–88.7)**	**87.10% (75.3–93.7)**	**80% (67.1–88.7)**	**0.91 ± 0.06**	**0.83 (0.71–0.95)**
KNN	74.51% (61.1–91.8)	64% (50.3–75.8)	80% (67.1–88.7)	70.97% (57.4–81.6)	71.11% (57.5–81.7)	0.79 ± 0.05	0.77 (0.63–0.91)

Performance metrics are reported with 95% confidence intervals. Mean cross-validation fold AUC values are presented together with their standard deviations to reflect variability across folds in the nested cross-validation procedure. Pooled out-of-fold AUC values summarize overall discriminative performance across all cross-validation iterations. Bold values represent the best-performing machine learning model.

**Figure 6:**
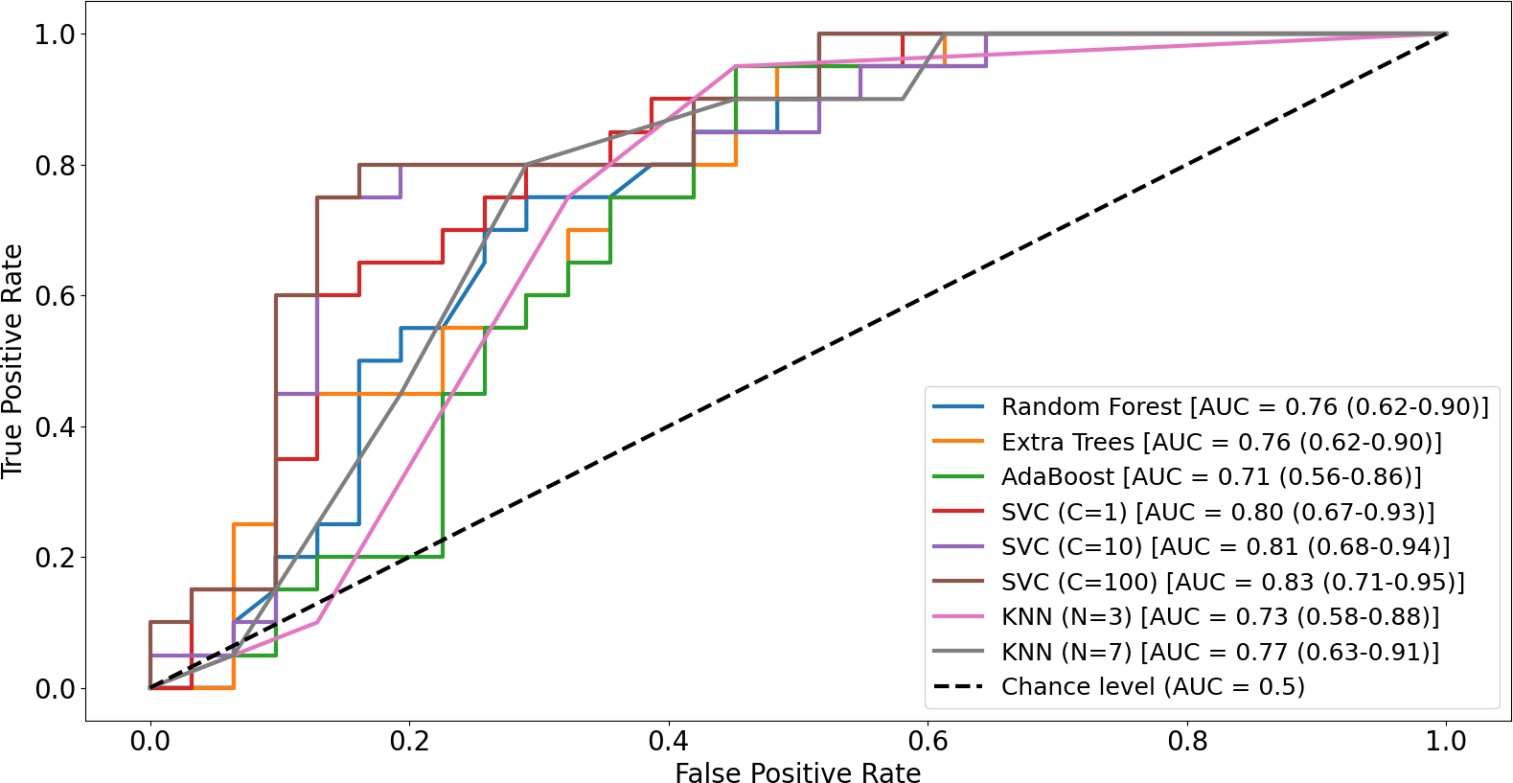
Receiver operating characteristic (ROC) curves illustrating the classification performance of the tested machine learning models in distinguishing the hyperactivity-risk group from the non-hyperactive comparison group. Curves are shown for Random Forest (RF), Extra Trees (XTR), AdaBoost (ADA), support vector machine (SVM), and k-nearest neighbors (KNN) classifiers. The diagonal dashed line represents chance-level performance (AUC = 0.50). Reported AUC values and 95% confidence intervals are based on pooled out-of-fold predictions across cross-validation folds.

The area under the curve is calculated both as mean AUC per each cross-validation fold, and as pooled out-of fold AUC. Since AUC is a nonlinear rank-based statistic, the mean of fold-wise AUCs differ from the AUC computed from pooled out-of-fold predictions. To that end, they are reported separately, where the mean CV-fold AUC values are presented in mean ± standard deviation format, while the pooled out-of-fold AUC values are reported along with the corresponding 95% confidence intervals using the Hanley and McNeil (1982) normal approximation based on pooled outer-fold predictions (N_1_ = 20 ADHD; N_2_ = 31 non-ADHD) (57). Since multiple variations of SVM and KNN were tested, the most optimal variation is represented here. See **Figure 6** for ROC curves for all tested classifiers, including each variation of SVM and KNN.

We trained an auxiliary model including only age and sex and evaluated its performance using the same cross-validation framework as the primary models to assess the potential influence of basic demographic variables. This demographic-only model, which again employed a support vector machine as the classifier, showed limited discriminative ability, with an accuracy of 58.82% (95% CI: 45.2-71.2), a mean CV-fold AUC of 0.59 ± 0.11 and a pooled out-of-fold AUC of 0.42 (95% CI: 0.26-0.58). These results show substantially lower performance compared to the primary models detailed previously. Together, these findings are consistent with the univariate analyses and indicate that age and sex contribute minimal predictive value in this sample (51 participants), suggesting that the observed model performance is not driven by basic demographic differences.

## Discussion

### Principal findings

This study suggests that movement-based behavioral features extracted from point-of-view (POV) video recordings during a standardized, naturalistic classroom interaction may help differentiate preschool children at risk for hyperactivity from their non-hyperactive peers. To our knowledge, this is the first study to employ teacher-worn POV video recordings combined with automated pose estimation to objectively quantify hyperactivity-related motor behavior in preschool-aged children within an ecologically valid educational context. Despite the two groups being comparable in age and sex, significant differences emerged across multiple body regions, including head, upper limb, and lower limb movement indices, as well as in overall global motor activity. Together, these findings suggest that POV-based, vision-driven assessment approaches hold promise as scalable and low-burden tools for supporting early screening of hyperactivity in preschool settings.

In addition to the observed group-level differences, machine learning analyses yielded complementary evidence suggesting that movement-based behavioral features may carry discriminative information. Among the evaluated classifiers, the support vector machine (SVM) achieved the strongest performance, yielding an overall accuracy of 84.31%, an F1 score of 80%, and an area under the ROC curve of 0.83. These findings indicate that region-specific motor activity patterns extracted from brief, naturalistic point-of-view (POV) recordings may help differentiate preschool children at risk for ADHD-related hyperactivity from their non-hyperactive peers. Importantly, this level of classification performance was obtained using a low-burden, ecologically valid assessment paradigm, highlighting the potential of pose-based behavioral markers to complement traditional symptom-based evaluations in early childhood.

### Comparison with prior work

Hyperactivity is one of the core and most developmentally salient features of Attention-Deficit/Hyperactivity Disorder (ADHD) (1). In response to the need for more objective assessment approaches, a growing body of research has explored the use of motion-based technologies to quantify hyperactivity-related behavior (58). Prior studies have employed a range of sensor-based and physiological measurement tools, including wearable motion sensors (59), accelerometers (25), IMT devices (27), multimodal platforms combining motion sensors with neurophysiological measures such as EEG (60) to capture motor activity patterns associated with ADHD. While these approaches have demonstrated promising associations with hyperactivity severity, they often require specialized equipment, prolonged monitoring periods, or active task engagement, which may limit feasibility and ecological validity in preschool and classroom settings.

More recently, immersive and technology-enhanced assessment paradigms have been introduced to address some of these limitations. For example, Oh et al. (2024) developed a diagnostic tool, AttnKare-D, which uses Virtual Reality (VR) and Artificial Intelligence (AI) to analyze behavioral data collected from children as they performed a series of everyday cognitive and behavioral tasks in a simulated VR environment. These tasks, such as organizing a room, packing a backpack, and planning a schedule, were designed to assess attention, hyperactivity, and impulsivity in contexts mimicking real-life situations. The analysis revealed that children with ADHD exhibited more frequent and prolonged movements in irrelevant spaces, higher frequencies of touching distracting objects, and more impulsive verbal responses compared to typically developing children. The AI model, which translated this multi-faceted behavioral data into a score based on the 18 DSM-5 ADHD symptoms, achieved an area under the curve (AUC) of 0.893 when compared to diagnoses made by child and adolescent psychiatrists, with a sensitivity of 80% and a specificity of 100% at its optimal cut-off score (23).

Building on VR-based assessment frameworks, immersive virtual reality paradigms have further incorporated eye-tracking technologies to obtain objective indices of attentional functioning in ADHD. Merzon et al. (2022) proposed an objective, technology-assisted diagnostic approach based on virtual reality (VR) and eye tracking to quantify attentional and executive function patterns in children with ADHD within an ecologically valid setting. Using a head-mounted display with an integrated 90 Hz eye tracker, they collected gaze data while participants performed EPELI (Executive Performance in Everyday Living), a VR task simulating everyday activities. The ADHD group exhibited significantly longer fixation durations, shorter saccade durations, and reduced saccade amplitudes compared to typically developing controls. A support vector machine (SVM) classifier trained solely on eye movement features achieved an AUC of 0.92, significantly outperforming classifiers based only on traditional task performance measures or on eye movements from a conventional visual search task (60).

In parallel, clinically scalable and unobtrusive device-based approaches have been developed to objectively capture hyperactivity through direct measurement of movement during routine clinical encounters. Chang et al. (2023) proposed an objective, device-based diagnostic method utilizing a smart chair embedded with piezoelectric material to quantify movement patterns in children with ADHD during clinical consultations. The study enrolled 31 children with ADHD and 31 age- and sex-matched controls, who were assessed while seated on the chair during routine outpatient visits. Movement signals were analyzed using variance (Var), zero-crossing rate (ZCR), and high-energy rate (HER) metrics. Results demonstrated that all three movement indices were significantly higher in the ADHD group compared to controls. A support vector machine (SVM) classifier achieved an area under the curve (AUC) of 98.00% using variance alone, indicating excellent discriminative power (61).

More closely aligned with the present study, recent work has demonstrated that non-contact, vision-based approaches can effectively quantify hyperactivity-related motor behavior in clinical contexts. Ouyang et al. (2024) proposed an objective, non-contact diagnostic framework based on skeleton detection and machine learning to quantify movement patterns in children with ADHD during outpatient consultations. Using OpenPose, they extracted 11 skeletal feature descriptors from 4–6 minute video recordings and analyzed movement variability in seated children. Among these features, the single descriptor thigh angle demonstrated the highest discriminative power, achieving an accuracy of 91.03%, sensitivity of 90.25%, specificity of 91.86%, and an AUC of 94.00%. By relying on a standard camera and preserving natural clinical interaction, this approach provides a practical and automated aid for distinguishing ADHD from non-ADHD cases based on objective motor activity (62).

When we consider our findings in the context of prior work, we observe both overlap and divergence in the movement features identified as most informative. While Ouyang et al. reported variability in thigh angle as a key skeletal feature, we found that distal lower and upper limb regions contributed most strongly to classification performance in our permutation-based feature importance analyses. Importantly, similar regions also showed stronger associations with teacher-rated hyperactivity severity, suggesting convergence between symptom-related behavioral variation and the features driving machine learning classification. At the same time, both studies point to the relevance of lower-limb involvement, indicating that leg-related movement may be an important component of hyperactivity-related motor behavior. We believe that differences in the specific regions highlighted across studies are likely related to contextual factors. Whereas Ouyang et al. examined seated behavior in structured outpatient settings, our paradigm involved children interacting in a familiar school environment during a naturalistic storytelling task. This may have encouraged more spontaneous fidgeting and distal limb movements and, in turn, influenced the relative importance of specific movement features.

Beyond movement-based markers, we note that a growing body of research suggests physiological signals reflecting autonomic regulation may also support ADHD prediction. Recent studies have shown that multimodal physiological features—such as electrodermal activity (EDA), heart-rate variability (HRV), and skin temperature—can contribute to machine-learning–based classification and may provide information that complements overt motor behavior (63). At the same time, we recognize that rapid advances in flexible, skin-conformal, and low-burden wearable technologies are improving the feasibility of comfortable, longer-term physiological monitoring in everyday environments (64). These developments may enable future multimodal screening frameworks that integrate behavioral and physiological signals.

A recent comprehensive review on ADHD detection approaches highlighted that objective measurement methods offer promising, cost-effective, and accessible tools for supporting ADHD assessment. Nevertheless, the review emphasized that such methods should be employed with caution, as only a subset of ADHD symptoms—primarily those related to hyperactivity—can be captured by motion-based measures, and therefore these approaches should not be considered sufficient as standalone diagnostic tools (65). In the preschool period, however, hyperactivity and impulsivity constitute the most prominent and developmentally salient manifestations of ADHD (10, 66). Accordingly, objective assessment strategies that prioritize ecologically valid measurement of motor activity may be especially well suited to preschool populations and yield more informative screening outcomes in early childhood ADHD.

Our study extends this growing body of work by addressing several key gaps in the existing literature. Whereas many prior objective assessment approaches have been developed and validated primarily in clinical settings, often involving school-aged children (typically 7 years and older), wearable devices, or highly structured experimental tasks, the present study focuses on preschool-aged children and captures behavior within a familiar educational environment. Previous methods frequently require children to wear sensors, engage with specialized equipment, or perform artificial tasks under test-like conditions, which may alter natural behavior and limit ecological validity—particularly in younger populations.

In contrast, our approach leverages a brief, everyday storytelling interaction conducted in the classroom, without requiring the child to wear any devices or attend a clinical setting. By embedding assessment within a naturalistic, socially meaningful activity and using teacher-worn point-of-view video to unobtrusively capture movement, the present method minimizes burden, reduces reactivity, and preserves children’s comfort. This design allows objective quantification of hyperactivity-related motor behavior under conditions that closely resemble daily preschool experiences. Such an approach is particularly relevant given that hyperactivity symptoms tend to predominate over inattention during the preschool years, underscoring the potential value of ecologically valid, movement-focused measures for early screening and risk identification in young children.

### Limitations

Several methodological considerations should be taken into account when interpreting the present findings. First, although computer-vision–based pose estimation provides an objective and scalable approach to quantifying motor activity, movement indices derived from monocular POV recordings may not perfectly capture children’s true motor behavior. Minor measurement inaccuracies related to tracking precision, occlusion, or camera perspective are unavoidable in naturalistic settings and may have introduced noise into the extracted features.

Second, the relatively modest sample size limits generalizability and may have reduced statistical power, particularly for detecting more subtle regional effects. Although nested cross-validation was employed to mitigate optimistic bias in the machine learning analyses, such bias cannot be fully excluded in small-sample settings. In addition, permutation-based feature importance estimates may be unstable under these conditions, and no external validation cohort was available. Replication in larger samples with independent test sets will therefore be essential to establish model stability and clinical relevance. Also, although multiple supervised classifiers were evaluated to provide a broad comparison, not all models are equally well suited to small-sample settings. In particular, instance-based and boosting approaches may exhibit greater variability under limited data conditions. Accordingly, results from these models were considered exploratory. For this reason, primary interpretation was focused on models with more stable and theoretically aligned behavior, such as support vector machines and tree-based ensembles.

Third, behavioral data were collected during a single brief one-on-one interaction, precluding assessment of intra-individual stability or test–retest reliability. The findings should therefore be interpreted as reflecting between-group differences at a single time point. Moreover, individual testing may not fully reflect children’s typical activity levels in classroom contexts, as some children may modulate their behavior during individualized interactions. Future studies incorporating repeated measurements and group-based classroom recordings would improve ecological validity.

Fourth, several feature-related considerations warrant caution. Because movement features were quantified as cumulative linear displacements in a pelvis-centered coordinate system, the resulting measures are inherently scale-dependent and may be influenced by anthropometric variability, such as limb length. Although the age range was relatively narrow, residual body-size effects cannot be excluded. In addition, root-centered representations emphasize relative segmental motion and may underrepresent large-scale postural translations involving concurrent pelvis displacement. Future work may benefit from incorporating normalization procedures, angular kinematics, or global motion descriptors.

Finally, potential confounding factors were not systematically assessed. Handedness was not formally measured and therefore could not be included as a covariate in the analyses. Because lateralized motor behavior in early childhood is influenced by emerging hand dominance, some left–right differences in movement indices may partly reflect individual dominance rather than hyperactivity-related motor patterns. In line with this, inter-regional movement analyses revealed strong bilateral correlations across homologous body segments (**Figure 4**), suggesting that apparent right-sided differences in feature importance likely reflect shared variance among correlated movement patterns rather than true lateralized motor dominance. Taken together, the pattern of results showed increased movement across multiple body regions and both sides of the body, which is more consistent with a generalized elevation in motor activity. Likewise, transient factors such as sleep quality, nutrition, or emotional state were not controlled. Future studies should include standardized measures of handedness and explicitly examine lateralized movement features, along with other transient influences such as sleep quality, nutrition, or emotional state, to better isolate symptom-related motor behavior.

## Conclusions

This study demonstrates that movement-based features extracted from teacher-worn point-of-view video recordings during a brief, naturalistic classroom interaction may help differentiate preschool children at risk for ADHD-related hyperactivity from their non-hyperactive peers. By combining ecologically valid data collection with automated pose estimation and machine learning, the proposed approach offers an objective, low-burden, and scalable method for quantifying hyperactivity in early childhood. Although not intended as a standalone diagnostic tool, this framework shows promise as a complementary screening approach that may support early identification efforts in preschool settings. Future studies with larger and more diverse samples are warranted to validate these findings and further explore the clinical and educational utility of POV-based behavioral assessment.

## Data Availability

The raw data supporting the conclusions of this article will be made available by the authors, without undue reservation.
