# Supplementary material for: Leveraging point-of-view camera and MediaPipe for objective hyperactivity assessment in preschool ADHD
Source: Front Psychiatry. 2026 Mar 4;17:1769322. doi: 10.3389/fpsyt.2026.1769322 (PMC12996202; doi:10.3389/fpsyt.2026.1769322)
Supplement: Supplementary file 1 [file Supplementaryfile1.docx]

**Appendix 1.** Story Stimulus (Six Segments) and Comprehension Questions

**Segment 1**

In the opening part of the story, a child and his grandmother begin a playful “travel game,” imagining a long journey together. They choose to travel slowly on a large ship and picture themselves passing by many islands during their voyage.
**Question 1:** What game are they playing?

**Segment 2**

As their imagined sea journey continues, they notice dolphins swimming nearby and racing alongside the ship. Later at night, the child looks out over the glowing water, and the grandmother explains that the shimmering light is caused by tiny sea organisms that illuminate the surface.
 **Question 2:** Which animals were racing in the sea?

**Segment 3**

In the next part of the story, the child suggests traveling by land. They imagine boarding a train in the early evening, watching the scenery pass quickly outside the window. During the ride, the grandmother sings a cheerful folk tune while the child holds her hand affectionately.
 **Question 3:** What is the grandmother singing on the train?

**Segment 4**

As night falls, they lie in their bunks while the train continues moving steadily. The grandmother tells a story, and the rhythmic movement makes the child sleepy. From the neighboring carriage, they hear the pleasant sound of an accordion playing. The child wonders whether they might continue their trip by bus later on.
 **Question 4:** What sound is coming from the next carriage?

**Segment 5**

Throughout the imagined journey, the child naps on his grandmother’s shoulder but wakes up during each stop. At one stop, she buys him a sweet treat and stretches her legs before continuing. They imagine traveling next by airplane and visualize a large, bright airport filled with blinking signs.
**Question 5:** What does the grandmother buy at the stop?

**Segment 6**

In the final part of the story, ongoing announcements make the child feel a bit overwhelmed, but boarding the airplane lifts their spirits as they picture themselves flying like birds. The segment ends with the child happily imagining planting flowers in the garden with his grandmother.
 **Question 6:** What do they plant in the garden?

**Note.** The story segments presented above are researcher-generated summaries adapted for use as standardized auditory stimuli. The original narrative, *Yolculuk Oyunu*, was published by the Turkish Ministry of National Education (MEB, 2023) and is not reproduced here verbatim.
